# Unusual Causes of Jaundice: A Diagnostic Challenge

**DOI:** 10.7759/cureus.49760

**Published:** 2023-11-30

**Authors:** Amiya Ranjan, Manish Kumar, Ashok Sunder, Zaid Nafe

**Affiliations:** 1 Department of Gastroenterology, Tata Main Hospital, Jamshedpur, IND; 2 Department of General Medicine, Tata Main Hospital, Jamshedpur, IND

**Keywords:** autoantibodies, hepatic failure, autoimmune hepatitis (aih), acute viral hepatitis, hepatitis a virus (hav)

## Abstract

This case report discusses a complex medical scenario involving a 25-year-old female patient initially diagnosed with acute hepatitis A virus (HAV) who later developed symptoms indicative of autoimmune hepatitis (AIH). The transition from uncomplicated HAV to impending subacute hepatic failure and autoimmune overlap syndrome highlights the importance of vigilant monitoring and a comprehensive diagnostic approach. The patient's medical evaluation revealed autoantibodies, elevated IgG levels, and liver biopsy findings consistent with steatohepatitis. Management included immunosuppressive therapy, resulting in a positive treatment response. The phenomenon of AIH following acute HAV infection, though rare, remains a subject of medical interest and presents diagnostic and therapeutic challenges. Further research and clinical experience are needed to develop effective strategies for these infrequent cases.

## Introduction

The fecal-oral mode of transmission for the hepatitis A virus (HAV) is a key contributor to acute viral hepatitis, which in extreme cases can result in acute liver failure (ALF) and fatality [1-2]. Clinical signs and symptoms of HAV infection range from asymptomatic illness to ALF, and some individuals exhibit unusual characteristics such as recurrent hepatitis or protracted cholestatic hepatitis in addition to extrahepatic manifestations [3].

The very robust HAV can withstand a variety of environments, including low pH, drying, and detergents. Foods must be heated to exceed 85°C for one minute to inactivate HAV, or surfaces must be cleaned with household bleach diluted 1:100 for one minute [1]. Hepatitis A virus may survive the stomach's acidity when consumed by the fecal-oral route and finally reach the liver, where it multiplies in hepatocytes (liver cells). The virus is subsequently discharged into the bile and returned to the digestive system. After neutralization and elimination take place, it is finally eliminated by feces or reabsorbed into the liver in an enterohepatic cycle [4]. Patients get acute hepatitis symptoms and increased serum aspartate aminotransferase (AST)/alanine aminotransferase (ALT) after an incubation period of 15-50 days (mean: 30 days) following HAV infection. Therefore, the prodromal stage, which precedes symptoms or biochemical signs, is when the risk of transmission is greatest. Even smaller amounts of the virus are also released in the saliva [5].

Hepatitis A frequently develops acutely, with fever (18%-75%), malaise (52%-91%), nausea or vomiting (26%-87%), stomach pain (37%-65%), dark urine (28%-94%), and jaundice as the first symptoms. Less frequently, arthralgia, skin rash, diarrhea, or pruritus appear. The fever often subsides by the time the patient seeks medical attention [6-7]. Hepatomegaly (78%) and jaundice (40%-80%) are often found during physical examinations. According to test findings, there are high levels of total bilirubin (7-13 mg/dL on average), alkaline phosphatase (319-335 IU/L), and ALT (1952-3628 IU/L). Very high ALT levels (>10,000 IU/L) are occasionally found; however, this is not always a bad sign [1]. In the absence of hemolysis, a prolonged prothrombin time (PT) of 40% and high bilirubin levels suggest severe hepatitis with a possible risk for ALF. Hepatomegaly and gallbladder wall thickness greater than 3 mm (80%) with vascular heterogeneity, periportal tracking, and perihepatic lymph node enlargement (>7 mm in diameter) are radiological signs of hepatitis A [1].

Hepatitis A virus infection does not develop into chronic hepatitis and has a self-limiting course. However, recurrent hepatitis or protracted cholestasis lasting more than six months occurs in 10%-20% of individuals. Up to 12% of people experience relapsing hepatitis after the first form has resolved; however, it often manifests in a lesser manner. In 5%-7% of individuals, prolonged cholestasis-defined as a total bilirubin level greater than 5 mg/dL for more than four weeks presented as tiredness and pruritus. Age of infection and pre-existing liver conditions have an impact on the severity of hepatitis A. The prevalence of ALF, which affects 0.015%-0.5% of hepatitis A patients, is higher in patients who are older (>40-50 years old) and those who have underlying chronic liver illnesses with a low level of hepatic functional reserve. Acute kidney injury, acalculous cholecystitis, pancreatitis, pleural or pericardial effusion, hemolysis, hemophagocytosis, pure red-cell aplasia, acute reactive arthritis, skin rash, and neurological manifestations like mononeuritis, Guillain-Barré syndrome, and transverse myelitis are among the extrahepatic manifestations that have been reported [7].

Contrarily, autoimmune hepatitis (AIH) is a chronic liver disease in which the immune system unintentionally targets healthy liver cells for destruction. Over time, liver damage and inflammation may result from this persistent immunological reaction. Numerous clinical manifestations of AIH, such as tiredness, jaundice, stomach pain, and increased liver enzyme levels, have been reported [8]. Although it is very uncommon, there have been reports of a link between HAV and AIH. Here is how this overlap may take place: (1) Autoimmune response initiation: an acute HAV infection in certain people can cause the liver to develop an autoimmune reaction. This implies that even after the HAV infection has subsided, the immune system, which was originally reacting to HAV, may become dysregulated and begin targeting healthy liver cells. (2) Simultaneous presentation: Rarely, a patient may show signs of both autoimmune hepatitis and acute HAV infection at the same time. The clinical picture and therapeutic strategy may be hampered as a result. Autoimmune hepatitis has occasionally appeared after a person has recovered from an acute HAV infection. This implies that in susceptible people, the initial viral infection may possibly contribute to the development of autoimmune hepatitis [9].

Not everyone who has an acute HAV infection will go on to develop AIH, and this overlap is quite uncommon. Such cases might be difficult to diagnose and manage since they frequently require a multidisciplinary strategy comprising hepatologists and immunologists. A thorough assessment, which may include serological tests, liver biopsies, and other diagnostic procedures, may be required if there is a suspicion of AIH after HAV infection in order to confirm the diagnosis and choose the best course of action, which may include immunosuppressive drugs.

In this case report, a 25-year-old female patient who originally arrived with acute HAV subsequently acquired symptoms suggestive of AIH. The clinical course and diagnostic problems encountered are discussed. Also, the patient's path from a straightforward HAV diagnosis to imminent subacute hepatic failure and an autoimmune overlap syndrome, emphasizing the significance of prompt diagnosis and treatment are discussed.

## Case presentation

A 25-year-old female patient presented with a concerning clinical history marked by a progressively deepening jaundice, associated with poor appetite. Notably, there were no accompanying symptoms such as clay-colored stools, nor were there any skin manifestations like rash, petechiae, or ecchymoses except mild pruritus. In addition, she displayed no signs of abdominal distension, pedal edema, or other features indicative of hepatic decompensation. This presentation had initially occurred six weeks prior, leading to her admission to the hospital with a diagnosis of HAV. She tested negative for other viral hepatitis co-infections. The diagnosis of acute HAV was substantiated by the presence of a viral prodrome history, which included fever, vomiting, and abdominal pain. These prodromal symptoms had subsided without the need for intensive intervention. The diagnosis was further confirmed through liver function tests (LFT), imaging studies, and viral serology. During her initial hospital stay, her condition showed significant clinical improvement, accompanied by a resolution of abnormal liver function markers. Consequently, she was discharged with appropriate medications aimed at alleviating her symptoms and promoting her recovery. However, the clinical course took an unexpected turn on follow-up six weeks after discharge. The patient experienced a worsening of jaundice with no presence of ascites, pruritis, or bleeding manifestations. However, notable deterioration in her liver function tests (Table 1) suggested a progression toward impending subacute hepatic failure.

**Table 1:** Liver function tests on different dates AST: aspartate aminotransferase; ALT: alanine transaminase; ALP: alkaline phosphatase

	Reference value	15^th^ May 2023	14^th^ June 2023	29^th^ June 2023	4^th^ July 2023
Total bilirubin	0.2-1.0 mg/dl	6.68 mg/dl	7.25 mg/dl	17.31 mg/dl	8.98 mg/dl
Direct bilirubin	0.1-0.5 mg/dl	1.36 mg/dl	6.41 mg/dl	13.79 mg/dl	7.57 mg/dl
Indirect bilirubin	0.1-0.7 mg/dl	5.32 mg/dl	0.84 mg/dl	3.52 mg/dl	1.41 mg/dl
AST	00-35 U/L	943.00 U/L	1311.20 U/L	910.40 U/L	374.50 U/L
ALT	00-45 U/L	515.50 U/L	877.20 U/L	458.80 U/L	33.30 U/L
ALP	53-141 U/L	289.30 U/L	290.32 U/L	137.90 U/L	114.60 U/L

Coagulopathy began to manifest, though there were no associated signs of ascites or bleeding. There was no significant medical history, no history of substance addiction, and a non-contributory family history. Clinical examination revealed a deep icterus, consistent with the progression of jaundice. An abdominal examination demonstrated mild hepatomegaly, but no stigmata of chronic liver disease were evident, nor were there any signs of hepatic decompensation. A thorough evaluation of other body systems yielded results within normal limits, without any abnormal findings.

This case presents a complex clinical scenario characterized by an initial diagnosis of acute HAV with subsequent complications, including impending subacute hepatic failure and coagulopathy. The absence of typical signs of hepatic decompensation, along with the unexpected progression of the disease, underscores the importance of ongoing clinical monitoring and a comprehensive diagnostic approach in such cases.

The patient's medical evaluation yielded the following findings: (1) Autoantibodies (antinuclear antibodies (ANA)) were positive with a titer of 1:100. The presence of autoantibodies, specifically a positive ANA test with a titer of 1:100, suggests an immune system response against the body's own cell nuclei. Antinuclear antibody positivity can be associated with autoimmune disorders. (2) Raised IgG levels in the blood that indicated an increase in a specific type of antibody. Elevated IgG can also be seen in autoimmune conditions. (3) Tests for cytomegalovirus (CMV), herpes simplex virus (HSV), Epstein-Barr virus (EBV), and varicella-zoster virus (VZV) were negative. (4) Wilson's workup was negative. (5) The USG of the whole abdomen was normal. (6) A USG Doppler performed for the hepatic-splenic-portal axis was normal. (7) Upper GI endoscopy revealed a normal study.

**Figure 1:**
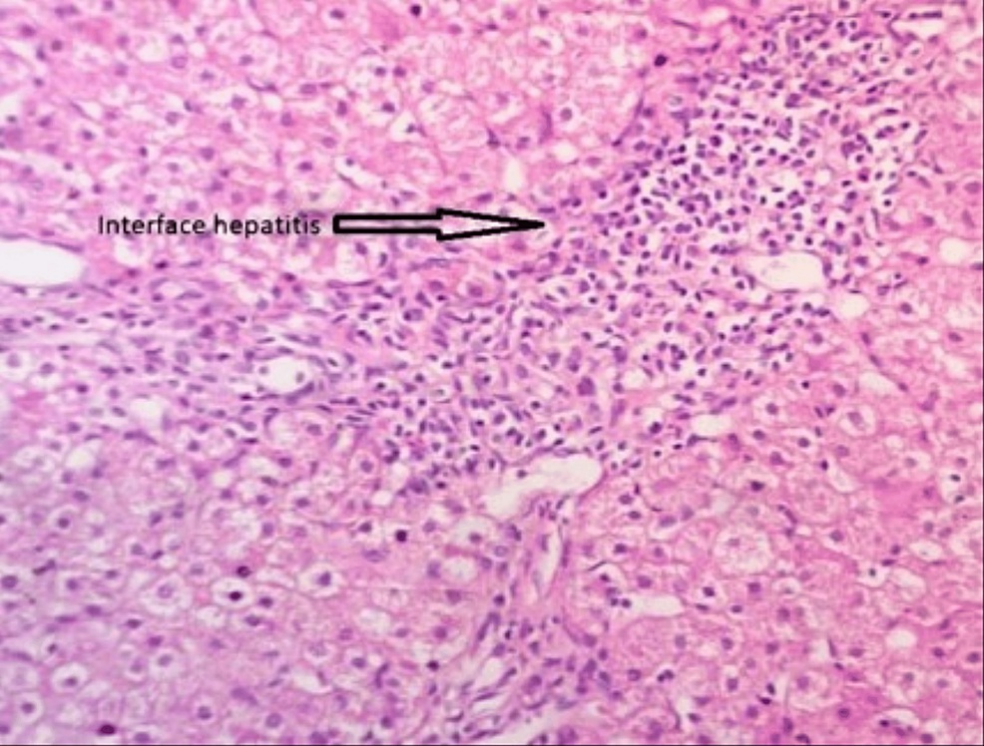
The histology of autoimmune hepatitis shows interphase hepatitis with periportal inflammation dominated by lymphocytes.

The microscopic examination of the specimen-liver biopsy section showed mild architectural changes with steatosis, focal lobular inflammation, portal and periportal inflammation, and dilated central veins. Inflammatory cells were comprised of lymphoplasmacytes with neutrophils and a few eosinophils. Mild focal fibrosis was seen. There was no evidence of granuloma or malignancy. Features were suggestive of steatohepatitis (Figure 1).

Management

The patient's treatment began with steroids, followed by a gradual tapering of the dosage. Azathioprine was introduced once the bilirubin levels had normalized, likely following thiopurine S-methyltransferase (TPMT) enzyme testing to determine the appropriate dose. The patient is currently asymptomatic, and her liver function tests are stable (Table 2), suggesting a positive response to the treatment plan.

**Table 2:** Liver function tests after treatment AST: aspartate aminotransferase; ALT: alanine transaminase; ALP: alkaline phosphatase

	Reference value	31^st^ July 2023	25^th^ August 2023
Total bilirubin	0.2-1.0 mg/dl	1.82 mg/dl	0.72 mg/dl
Direct bilirubin	0.1-0.5 mg/dl	0.71 mg/dl	0.32 mg/dl
Indirect bilirubin	0.1-0.7 mg/dl	1.11 mg/dl	0.40 mg/dl
AST	00-35 U/L	27.20 U/L	26.30 U/L
ALT	00-45 U/L	34.60 U/L	27.70 U/L
ALP	53-141 U/L	83.20 U/L	105.40 U/L

Continued monitoring and follow-up care will be essential to ensure the ongoing management of the autoimmune condition.

## Discussion

The phenomenon of AIH occurring after an acute HAV infection is a rare but intriguing medical condition. While it remains relatively uncommon, it has been reported in the medical literature, leading to ongoing discussions and challenges in its diagnosis and management.

The proposed pathophysiology of AIH following acute HAV infection revolves around the immune system's response to the virus. Hepatitis A virus infection could potentially disrupt the balance of the immune system, particularly affecting the suppressor-inducing T cells. These cells play a crucial role in regulating the immune response and preventing autoimmune reactions. Disruption in their function might lead to an autoimmune response characterized by the production of autoantibodies [10].

Autoimmune hepatitis is characterized by an immune system attack on the liver, often mediated by autoantibodies. In the context of HAV infection, it is hypothesized that the altered immune response triggered by the virus may contribute to the development of AIH [11]. However, the precise mechanisms behind this phenomenon are not fully understood and require further research.

Between the first diagnosis of acute HAV infection and the workup for the evaluation of AIH, there is a noticeable temporal lag in published data. With fluctuations, the stated mean duration is around 37 days. This lag period raises the possibility that AIH may not develop immediately following HAV infection but may take some time to manifest or become clinically obvious [12].

Immunosuppressive medications, such as corticosteroids or other medicines, were probably part of the treatment plans for these patients to reduce the autoimmune reaction. However, based on the characteristics of each patient and the degree of liver involvement, the kind of treatment and its duration may differ [13].

Hepatologists and physicians continue to have a considerable number of hurdles when trying to diagnose AIH in the context of HAV infection. This condition has a number of restrictions and difficulties, including the rare occurrence of a delay between HAV infection and the onset of AIH, which makes identification more difficult since patients may first exhibit signs and symptoms of acute HAV infection before AIH becomes apparent. A treatment conundrum might arise when deciding whether and how to begin immunosuppressive medication in the presence of an HAV infection. Immunosuppressive therapy has the danger of escalating the viral infection or making it more difficult for it to resolve.

Absence of guidelines

There are no established standards for the care of this illness since it is so uncommon. Clinicians must make individualized treatment decisions based on available evidence and the patient's specific clinical presentation [10].

## Conclusions

The occurrence of AIH following an acute HAV infection is a rare and challenging medical condition. While there is a suggested pathophysiological link between the two, the precise mechanisms are not well understood. The management of AIH in the setting of HAV infection poses clinical dilemmas, and further research and clinical experience are needed to develop effective diagnostic and treatment strategies for these rare cases.
